# Early Ahmed Glaucoma Valve Implantation after Penetrating Keratoplasty Leads to Better Outcomes in an Asian Population with Preexisting Glaucoma

**DOI:** 10.1371/journal.pone.0037867

**Published:** 2012-05-21

**Authors:** Ming-Cheng Tai, Yi-Hao Chen, Jen-Hao Cheng, Chang-Min Liang, Jiann-Torng Chen, Ching-Long Chen, Da-Wen Lu

**Affiliations:** 1 Department of Ophthalmology, Tri-Service General Hospital, National Defense Medical Center, Taipei, Taiwan; 2 Graduate Institute of Medical Science, National Defense Medical Center, Taipei, Taiwan; Oregon Health & Science University, United States of America

## Abstract

**Background:**

To evaluate the efficacy of Ahmed Glaucoma Valve (AGV) surgery and the optimal interval between penetrating keratoplasty (PKP) and AGV implantation in a population of Asian patients with preexisting glaucoma who underwent PKP.

**Methodology/Principal Findings:**

In total, 45 eyes of 45 patients were included in this retrospective chart review. The final intraocular pressures (IOPs), graft survival rate, and changes in visual acuity were assessed to evaluate the outcomes of AGV implantations in eyes in which AGV implantation occurred within 1 month of post-PKP IOP elevation (Group 1) and in eyes in which AGV implantation took place more than 1 month after the post-PKP IOP evaluation (Group 2). Factors that were associated with graft failure were analyzed, and the overall patterns of complications were reviewed. By their final follow-up visits, 58% of the patients had been successfully treated for glaucoma. After the operation, there were no statistically significant differences between the groups with respect to graft survival (p = 0.98), but significant differences for IOP control (p = 0.049) and the maintenance of visual acuity (VA) (p<0.05) were observed. One year after surgery, the success rates of IOP control in Group 1 and Group 2 were 80% and 46.7%, respectively, and these rates fell to 70% and 37.3%, respectively, by 2 years. Factors that were associated with a high risk of AGV failure were a diagnosis of preexisting angle-closure glaucoma, a history of previous PKP, and a preoperative IOP that was >21 mm Hg. The most common surgical complication, aside from graft failure, was hyphema.

**Conclusions/Significance:**

Early AGV implantation results in a higher probability of AGV survival and a better VA outcome without increasing the risk of corneal graft failure as a result of post-PKP glaucoma drainage tube implantation.

## Introduction

The presence of glaucoma following a penetrating keratoplasty (PKP) procedure is the second most common cause of corneal graft failure [1]. Some patients who have corneal pathology that requires PKP have preexisting glaucoma; Reinhard et al [2] estimated that the 3-year graft survival rate in these patients is approximately 71%, as opposed to an 89% survival rate in patients with no history of glaucoma. The implantation of glaucoma drainage devices (GDDs) has therefore played an important role in the surgical treatment of glaucoma in patients who have undergone PKPs [3]. Several reports have shown that using GDDs as a method of treating glaucoma, as is the case with Ahmed valve (AGV) implantation, is an effective method of controlling intraocular pressure (IOP) in glaucoma patients. In a number of studies, 50–80% of the patients experienced post-operative corneal graft rejections that affected their visual acuities (VAs) [4]–[6]. At present, there is no consensus regarding the amount of time between PKP and AGV implantation that is optimal for controlling IOP, improving graft survival, and preserving VA in patients with preexisting glaucoma. Moreover, there have been no studies that compare the surgical outcomes of patients who received AGV implantations at various intervals after undergoing PKPs.

The purpose of the present study was to evaluate the procedure of using AGV implantation to control preexisting glaucoma following PKP in patients from an Asian population. This study compares the IOP, corneal graft, and visual acuity outcomes of post-PKP patients who received AGV implantation either within 1 month of post-PKP IOP elevation or more than 1 month after IOP elevation. The outcome measures were monitored for as long as 2 years after PKP. In addition, the factors that were associated with AGV failure in these patients, and the overall complications that they experienced were also analyzed. Finally, we found that earlier AGV implantation following post-PKP IOP elevation in patients with preexisting glaucoma and who underwent PKPs improved the probability of tube survival and preserved VA without increasing the likelihood of corneal graft failure.

## Methods

### Objectives

AGV implantation is a suitable treatment method for various types of glaucoma, including the treatment of glaucoma that is associated with undergoing a PKP procedure. For the most part, the present study aimed to evaluate whether more aggressive glaucoma treatment (early AGV implantation) was of greater benefit to patients with preexisting glaucoma who had undergone PKP. The study also placed a minor focus on evaluating the risk of AGV failure in these patients.

### Participants

We reviewed the medical records of patients with preexisting glaucoma and significant corneal disease that required PKP who were subsequently treated with AGV implantation at the Department of Ophthalmology of the Tri-Service General Hospital, Taipei, Taiwan, between January 2000 and December 2010. In total, 73 cases were reviewed, and 28 cases were excluded because the medical records were incomplete. A total of 45 eyes of 45 patients were included. All of the AGV implantation surgeries were performed by the corresponding author, and no other GDDs were used during the study period. Prospective patients who were not able to attend follow-up visits during an extended post-operative period were also excluded.

The patients were divided into 2 groups: Group 1 included patients in whom AGV implantation was performed within 1 month of determining the presence of persistent IOP elevation (measured IOP of ≥21 mm Hg at three successive visits), and Group 2 included patients in whom AGV implantation was performed more than 1 month after a persistent IOP elevation was established. Our hospital is a tertiary referral center, so most of the patients who were recruited for participation in our study were referred from other hospitals. To ensure corneal graft survival, we performed surgical interventions as soon as was possible. The criteria that were used in grouping these patients were therefore based on the information contained in the referral documents that we received when the patient was referred to our hospital.

### Description of Procedures

Pre- and postoperative patient demographics and clinical characteristics, including their ages, genders, IOP measurements (using a Goldmann applanation tonometer), corneal diagnoses, types of preexisting glaucoma and use of antiglaucoma medications, were documented and subjected to statistical analysis.

A similar surgical technique was used to perform AGV implantation in all patients. Under peribulbar anesthesia, we created a fornix-based conjunctival flap in the superotemporal quadrant between 2 adjacent recti muscles. After creating a 3×3 mm triangular scleral flap, the AGV (model S2 with a 185 mm^2^ polypropylene plate; New World Medical, Rancho Cucamonga, CA, USA) was irrigated with balanced saline solution (BSS; Alcon, Fort Worth, TX, USA) to prime the valve mechanism. The polypropylene body of the implant was placed 8 mm posterior to the corneoscleral limbus and was sutured to the sclera with an 8–0 prolene suture (Ethicon Inc., Somerville, NJ, USA). The tube of the AGV implant was then trimmed so that the bevel of it faced the corneal endothelial surface and was subsequently inserted into the anterior chamber through a needle track that had been made with a 23-gauge needle. A scleral patch graft from a human donor was placed on the tube so that the anterior edge was adjacent to the limbus and was then sutured to the sclera with an 8–0 prolene suture. After the implant and graft had been inserted, 0.5 cc of a viscoelastic solution (Healon GV^®^; Advanced Medical Optics, Santa Ana, CA, USA) was injected into the anterior chamber to avoid early hypotony. Finally, the conjunctiva was sutured to the limbus, and the eye received a subconjunctival injection of steroids and antibiotics. No adjunctive metabolites were used.

After the operation, topical eye drops containing 0.3% ofloxacin (Tarvid, Santen, Osaka, Japan) and 1% prednisolone acetate (EconoPred Plus, Alcon, Texas, USA) were prescribed, and their use was tapered slowly over a period of 4–8 weeks. Antiglaucoma medication prescriptions were adjusted on the basis of both the IOP and the clinical status of the eye that had received the implant. Patients were examined at a specific series of postoperative intervals (1 day, 1 week, and 1 month after surgery) and every 3 months thereafter for a total follow-up period of 2 years. Slit-lamp examinations were performed, and VA, IOP, and any surgical complications were assessed at each follow-up visit.

The outcome variable that we used to measure the success of AGV survival was postoperative IOP control after AGV implantation. Complete success was defined as having a final IOP that was <21 mm Hg, >6 mm Hg, and accompanied by a pressure reduction of at least 20% relative to pre-surgery levels in the absence of any loss of light perception, the need for any additional antiglaucoma medication, or AGV implant removal. Partial success was defined as a final IOP that was <21 mm Hg, >6 mm Hg, and accompanied by a pressure reduction of at least 20% relative to pre-surgery levels in conjunction with a need for additional antiglaucoma medication. Patients with IOPs that were ≥21 mm Hg or that were ≤6 mm Hg were given treatment that attempted to lower or raise their IOPs, respectively, and they were re-examined within several days to a week. Because these patients required more frequent postoperative examinations than patients in whom the AGV implant had been partially or completely successful, the results of their additional examinations were averaged to generate statistics for a single time frame. Neither success nor failure was defined until at least 2 consecutive examinations after the 3- to 6-month time frame had taken place. Success and failure of graft clarity survival were defined as follows: success was defined as the corneal graft remaining clear, and failure was determined by the presence of corneal graft decomposition. Additional outcome parameters included changes in visual acuity, operative complications, and postoperative complications.

### Ethics

The study followed the principles that were established in the Declaration of Helsinki and was approved by the Institutional Review Board of the hospital.

### Statistical methods

The Mann-Whitney *U* test and Fisher's exact test were used to compare non-parametric continuous and categorical variables, respectively, within the groups. Differences between the preoperative IOPs and the IOPs that were measured at each follow-up examination were analyzed using Wilcoxon signed-rank tests. Means were used to describe non-parametric data, and categorical data were represented by numbers and percentages. Kaplan-Meier life-table analysis was used to calculate IOP and graft survival curves. The following factors that may have influenced the rates of AGV failure were assessed in a logistic regression model: age, gender, diagnosis of glaucoma, total number of antiglaucoma medications, lens status, total number of previous PKPs, and postoperative IOP. All statistical assessments were two-tailed, and a P-value of P≤0.05 was considered statistically significant. Statistical analyses were performed using version 15.0 of the SPSS statistical software package (SPSS Inc., Chicago, IL, USA).

## Results

Demographic and preoperative characteristics of Groups 1 and 2 are listed in Table 1. Data that were collected include the ages, genders, preoperative IOPs, mean numbers of PKPs, types of preexisting glaucoma and corneal disease diagnoses. The most common type of glaucoma that was diagnosed in both groups was chronic angle-closure glaucoma (55% and 60% of patients in Groups 1 and 2, respectively). The major corneal disorder in both groups was pseudophakic bullous keratopathy (60% and 52% in Groups 1 and 2, respectively). The average follow-up period for patients in Group 1 and Group 2 was 22.4 months (SD, 11.3) and 17.8 months (SD, 12.0), respectively. The average time between PKP and AGV implantation was 74.5 days (SD, 40.5) in the Group 1 patients and 111.4 days (SD, 43.4) in the Group 2 patients (*P*<0.05).

**Table 1:** Preoperative characteristics of study patients in both groups.

	Group 1	Group 2	P-value
Patients (Number)	20	25	0.063
Age (Mean)	62.8	59.5	0.77
Female, No. (%)	11 (55%)	15 (60%)	0.38
Preoperative IOP (mmHg), mean (SD)	30.3 (5.28)	27 (6.25)	0.057
Duration between PK and persistent IOP elevation (days), mean (SD)	56.3 (39.9)	58.0 (42.3)	0.93
Preoperative antiglaucoma medications, mean (SD)	2.3 (1.3)	1.9 (0.9)	0.03^a^
Type of preexisting glaucoma diagnosis, No. (%)			0.43
Primary open angle	4 (20)	5 (20)	
Chronic angle closure	11 (55)	15 (60)	
Secondary (trauma, uveitis)	3 (15)	4 (16)	
Other	3 (15)	1 (4)	
Type of corneal diagnosis, No. (%)			0.58
Pseudophakic bullous keratopathy	12 (60)	13 (52)	
Failed PK	5 (25)	7 (28)	
Other	3 (15)	5 (20)	

Abbreviations: IOP  =  intraocular pressure; SD  =  standard deviation; ^a^Denotes statistical significance.

**Figure 1:**
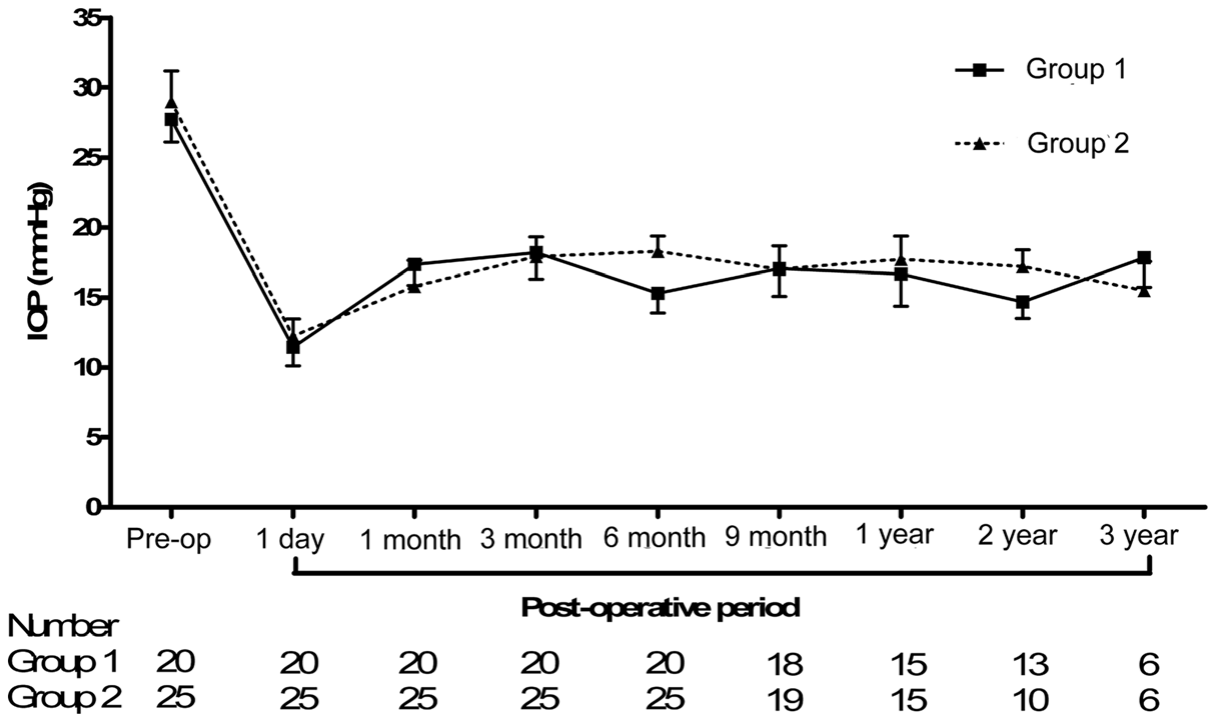
Pre- and postoperative intraocular pressures following Ahmed glaucoma valve implantation surgery in Group 1 and Group 2 over time. A marked decrease in the median IOP relative to the baseline IOP was noted during each postoperative period.

Figure 1 shows the IOP data that were obtained during the preoperative examination and postoperative follow-up periods in patients from Groups 1 and 2. The mean preoperative IOP of Group 1 patients was 27.8 mm Hg (SD, 7.3), and the mean preoperative IOP of the Group 2 patients was 29.0 mm Hg (SD, 10.9). After the operation, the mean IOPs of both groups decreased significantly at postoperative day 1, month 1, month 3, month 6, month 9, year 1, year 2, and year 3 (*P*<0.05). The mean IOP at the final follow-up examination had also decreased significantly in both groups; it reached a final value of 14.9 mm Hg (SD, 4.4) in group 1 (*P*<0.001) and a final value of 15.0 mm Hg (SD, 4.1) in Group 2 (*P*<0.001).

**Figure 2:**
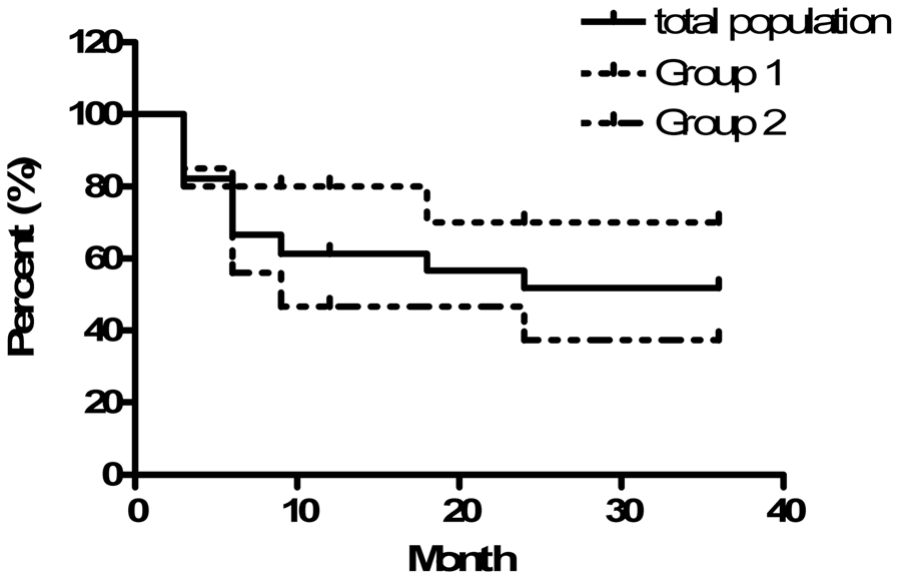
Kaplan-Meier life-table analysis showing the cumulative probabilities of IOP control at 1 year and 2 years post-PKP in Group 1, Group 2, and the entire sample population following Ahmed glaucoma valve implantation either with or without the use of antiglaucoma medications. (Log-Rank test  = 0.049).

The rate of completely successful Ahmed valve implantation was 40.0% (18/45), and the partial success rate of AGV implantation was 17.8% (8/45) in all patients at the last visit. The Kaplan-Meier life-table analysis for AGV survival in the 2 groups is shown in Figure 2. The overall cumulative probability of success was 58.9% at 1 year after implantation and was 49.4% at 2 years after implantation. The probabilities of success in Group 1 and Group 2 were 80% and 46.7% at 1 year and 70% and 37.3% at 2 years, respectively. There was a statistically significant difference between the two groups with respect to final success rate of IOP reduction (log-rank test  = 0.049).

**Figure 3:**
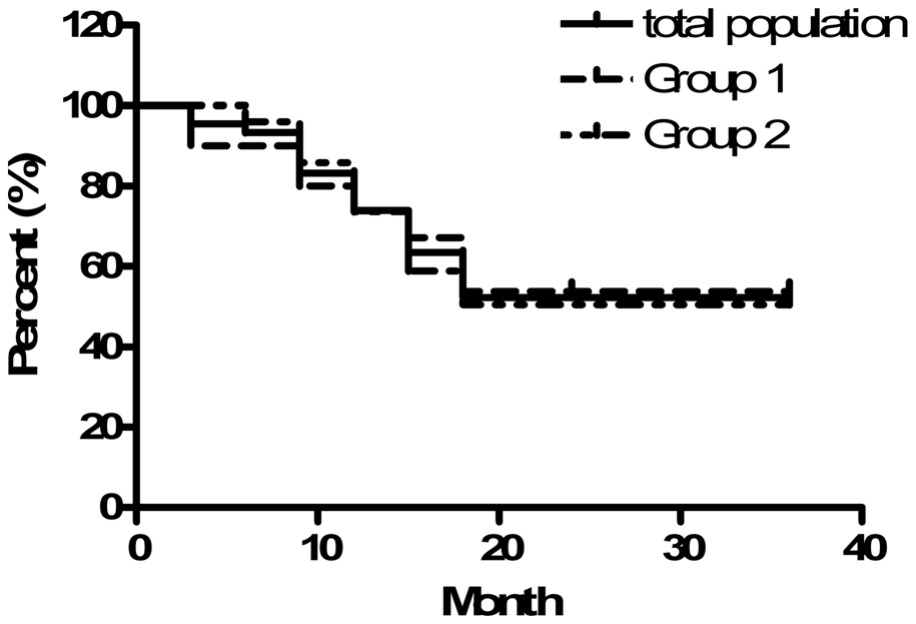
Kaplan-Meier life-table analysis showing the cumulative probabilities of graft survival at 1 year and 2 years post-PKP in Group 1, Group 2, and the entire sample population following Ahmed glaucoma valve implantation either with or without the use of antiglaucoma medications. (Log-Rank test  = 0.98).

The overall cumulative probabilities of corneal graft success were 74.0% and 52.2% at 1 and 2 years postoperatively, respectively (Figure 3). These probabilities were 73.8% and 73.6% at 1 year and 53.7% and 50.5% at 2 years in the Group 1 and Group 2 patients, respectively. There was no statistically significant difference between the two groups with respect to final rate of corneal graft survival (log-rank test  = 0.98).

**Figure 4:**
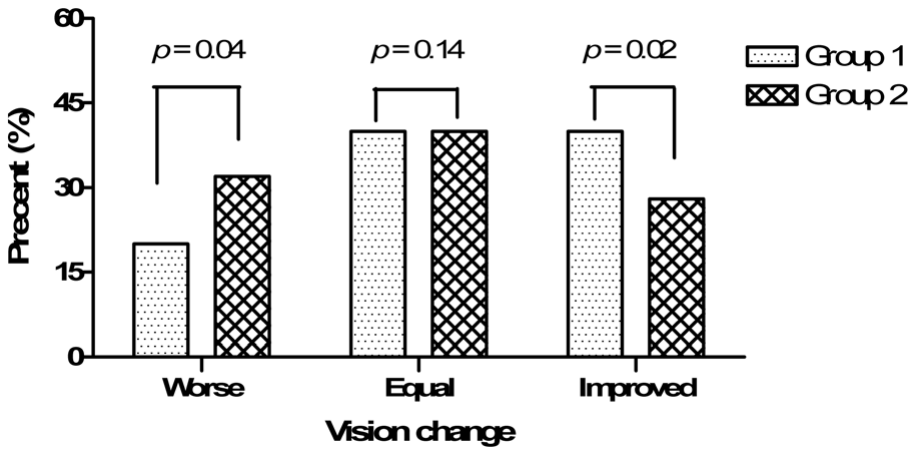
Visual acuity status at the final follow-up visit in Groups 1 and 2.

Figure 4 shows a comparison of the final changes in the visual acuities of the two groups. There was a significant difference in the final VAs of the “worsened” (p = 0.04, Fisher's exact test) and “improved” (p = 0.02) subgroups. However, there were no differences between either of these groups and the subgroup that experienced “no change.” In Group 1, 8 patients (40%) showed no change in VA, 4 patients (20%) showed a decline in VA, and 8 patients (40%) showed an improvement in VA. Similarly, 10 patients in Group 2 (40%) had no change in VA, 8 patients (32%) showed a decline in VA, and 7 patients (28%) showed an improvement in VA.

The average number of antiglaucoma medications that patients were using prior to AGV implantations was 2.3 (SD, 1.3) in Group 1 and 1.9 (SD, 0.9) in Group 2, and the difference between the numbers of medications taken by each group was significant (*P*<0.05). After undergoing various operations, the mean number of medications was 1.0 (SD, 1.1) in the Group 1 patients and 1.3 (SD, 1.2) in the Group 2 patients; the difference between the two groups was not significant.

As shown in Table 2, age, gender, diagnosis, lens status, number of previous PKPs and pre-operative IOP were analyzed as potential risk factors for AGV implantation failure. The Cox proportional hazards model indicated that the hazard ratio of AGV implant failure was significantly increased by having a diagnosis of preexisting glaucoma, the number of previous PKPs, and the pre-operative IOP after the AGV. The chronic angle-closure glaucoma patients appeared to be predisposed to higher rates of failure compared with other patients (OR, 3.55; 95% CI, 1.05–5.79; *P* = 0.034). In addition, having two or three previous PKPs was associated with an elevated risk of failure (ORs, 1.63 and 1.92, respectively; 95% CIs, 1.39–1.93 and 1.78–2.28, respectively; *P* = 0.042) as was an individual's preoperative IOP (OR, 3.01; 95% CI; 2.50–5.20; *P* = 0.02).

**Table 2:** Logistic regression analysis of risk factors for AGV failure at 2 years.

	Hazard Ratio	95% CI	P-value
Age (years)			0.054
<60	1.00	–	
≧60	1.03	(1.00, 1.05)	
Gender			0.494
Male	1.00	–	
Female	1.08	(0.55, 3.44)	
Diagnosis (glaucoma type)			0.034^a^
Primary open angle	1.00	–	
Chronic angle closure	3.55	(1.05, 5.79)	
Secondary (trauma, uveitis)	2.44	(0.84, 4.41)	
Lens status			0.064
Pseudophakia	1.00	–	
Aphakia	1.17	(0.77, 5.16)	
Phakia	1.08	(0.73, 4.98)	
Previous PKs			0.041^a^
One	1.00	–	
Two	1.63	(1.39, 1.93)	
Three	1.92	(1.78, 2.28)	
Postoperative IOP			0.02^a^
<21	1.00	–	
≧21	3.01	(2.5, 5.2)	

Abbreviations: CI  =  confidence interval; IOP  =  intraocular pressure; PK  =  penetrating keratoplasty; ^a^Denotes statistical significance.

The postoperative complications that occurred in patients in both groups are summarized in Table 3. The most frequent complications (in order of decreasing frequency) were as follows: corneal graft failure or rejection, hyphema, and the presence shallow anterior chamber.

**Table 3:** Postoperative complications in the 2 study groups.

	Group 1 (No., %)	Group 2 (No., %)
Shallow anterior chamber	2, 10	3, 12
Corneal graft failure or rejection	8, 40	8, 32
Serous choroidal detachment	1, 5	0, 0
Encapsulated bleb	1, 5	1, 4
Tube malposition	1, 5	1, 4
Diplopia	0, 0	1, 4
Hypotony	2, 10	2, 8
Fibrinous iridocyclitis	0, 0	1, 4
Hyphema	3, 15	3, 12

## Discussion

Ahmed glaucoma valve implantation often enables the successful control of refractory glaucoma in cases in which other surgical modalities are ineffective [7]–[12]. This undoubtedly poses a challenge to the surgical management of patients with preexisting glaucoma who have undergone PKPs. It has also been shown that pre-existing glaucoma is a risk factor for graft failure [13]. Although various types of GDDs have been effective in IOP control, many patients who received GDD implants have shown poor corneal graft outcomes with graft failure rates that ranged from 10 to 51% [3], [14]–[18]. Several studies have investigated the effect of the relative sequence of PK and GDD on graft survival. Rapuano et al [19] found evidence of a tendency toward decreased graft survival when a GDD was implanted after a patient had undergone PK. However, Coleman et al [6] found that there was no difference in outcomes when an AGV was implanted concurrently with or after a PK. Kwon et al [10] reported that eyes in which a GDD had been implanted prior to PK have a higher risk for graft failure than eyes in which GDDs were implanted concurrently with or after PKs. They also considered the fact that these patients tend to have severe glaucoma, which in turn could affect graft survival. In the case series that we reviewed in the present study, the total corneal graft survival rate was 52.2% at 2 years postoperatively, which is similar to the survival rate that has been reported in previous studies. Moreover, there was no statistically significant difference in the graft survival rates of the two groups. However, the success rate for controlling glaucoma was significantly higher among Group 1 patients compared with the success rate among Group 2 patients. These results demonstrate that early surgery can effectively improve the success rate in controlling glaucoma without inducing an increased risk of graft failure. In addition, several other mechanisms have been associated with a potentially higher risk of graft failure, such as excessive surgical time, multiple procedures, excessive postoperative inflammation, or early tube endothelial touch [10], [14]. Fortunately, these factors did not play especially strong roles in the clinical histories and outcomes of our patients. We performed anterior chamber injections of 0.5 cc of a viscoelastic solution (Healon GV^®^; Advanced Medical Optics, Santa Ana, CA, USA) and used AGV tubes of relatively short lengths to avoid complications such as early shallow anterior chamber and early tube endothelial touch after the suture in our patients.

Goldberg's study found that 71% of patients with pre-existing glaucoma developed increased IOPs early in the postoperative course following PKP [20]. In the present case series, AGV implantation succeeded in controlling glaucoma in 80% and 71% of patients at 1 and 2 years, respectively. Alvarenga et al [14] reported that eyes in which Ahmed valves were implanted had glaucoma control success rates of 74% and 63.1% at 1 and 2 years, respectively, which are higher than the success rates in our patients. This finding may be a result of the time at which the AGV was implanted. In our study, an aggressive therapeutic protocol in which an AGV was implanted within 1 month of the establishment of elevated IOP (Group 1) showed survival rates of AGV implantation that were similar to those of previous reports, whereas a less intensive treatment protocol in which AGV implantation occurred more than 1 month after an elevated IOP was established (Group 2) yielded an opposite result. Furthermore, the inclusion of eyes with different types of preexisting glaucoma may play a role in this issue. Our study differs from other studies that have been mentioned in that the majority of patients who were included in it had chronic angle-closure glaucoma. In general, AGV implantation has been thought to be relatively effective with respect to controlling glaucoma in different types of patients [21], [22], and neither glaucoma nor corneal diagnosis has been shown to influence the success of long-term glaucoma control with GDDs. However, our experience has shown the success rate of AGV implantation may be influenced by the type of glaucoma; for example, we have found evidence of poor outcomes of AGV implantation in controlling neovascular glaucoma [22]. Furthermore, our Cox regression analysis also showed that the patients with preexisting angle-closure glaucoma had an increased risk of AGV failure compared with other patients. Thus, we believe that the clinical characteristics of patients with different types of glaucoma may differ and could result in diverse outcomes.

Escalation of glaucoma therapy often immediately follows PKP in patients with a preexisting glaucoma condition [23] in which the rapid onset of IOP control could diminish the severity of optic nerve damage. In our study, a short latency between PKP and AGV implantation is related to the improved success rate of AGV implantation and to the preservation of VA. An increased duration of elevated IOP prior to Ahmed valve implantation may lead to greater inflammation that could cause further damage to the trabecular cells [24]. Therefore, earlier AGV implantation may preserve the residual functioning of trabecular meshwork by lessening the degree of trabecular cell death. In addition, we also found that patients who underwent AGV implantation shortly after PKP generally had better VA outcomes than patients for whom the interval between PKP and AGV implantation was longer. Although the visual field data to support this result were not available, we speculate that this result might be due to the lessening of optic damage that resulted from early IOP control.

The Cox regression analysis that we conducted showed that the type of glaucoma with which a patient had been diagnosed, the number of previous PKPs, and the duration of IOP elevation prior to AGV implantation were all associated with an increased risk of AGV failure. The majority of our patients had chronic angle-closure glaucoma, which was associated with a higher risk of AGV failure. No previous study has found evidence of an increased rate of AGV failure in angle-closure glaucoma. We suggest that this increase may result from having a narrow angle space that is occupied by a tube. The presence of the tube may easily cause anterior chamber inflammation due to friction against the angle walls. Moreover, inflammation in the anterior chamber may decrease the AGV success rate, which is the case in uveitic glaucoma [25]. Multiple previous PKPs can lead to an increased tendency to develop anterior chamber synechiae and corneal neovascularization [26], which may also result in a high risk of AGV failure. Preoperative IOP elevation may reflect a refractory disease status in which the disease cannot readily be controlled by drugs or by laser therapy. Thus, the elevated risk of AGV failure that has been observed in these patients was reasonable.

In our study, the most common early postoperative complication was hyphema, followed by a shallow anterior chamber. The incidence of hyphema following AGV implantation has been reported to occur in approximately 2%–20% of patients, and it typically resolves without surgical intervention [17], [21], [27], [28]. The incidence of a shallow anterior chamber following the implantation of an Ahmed valve has been reported to be 0–15% [17], [21], [27], [28]. Differences between these reports and our findings might be explained by the clinical statuses of our patients and by the particular variation in the surgical technique that we used in which a viscoelastic solution was injected immediately following valve implantation to prevent early hypotony or choroidal effusions. Fortunately, this condition typically resolves spontaneously without additional surgery. No serious complications that involved VA losses or blindness occurred among our patients.

There are several limitations to our study. The retrospective design with variable follow-up intervals may result in certain patient selection biases, and the inclusion of patients with various glaucoma diagnoses resulted in a relatively small sample size. However, it is difficult to conduct a prospective and randomized trial because of ethical concerns and because the amount of time that elapses between PKP and AGV implantation cannot be masked. Moreover, the continual availability of new drugs makes it impossible to control for type of ocular medications. The VAs of some patients might be influenced by both corneal pathology and preexisting glaucoma, which in turn may also result in certain biases in the assessment of VA. Antiglaucoma drug use was more prominent among patients in group 1, which may reflect a generally greater severity of glaucoma in this group. The elevated severity of glaucoma may have proceeded to interfere with the success rate of AGV implantation. Another possible reason for some of the observed inter-group differences is that the patients in group 1 were referred by an aggressive corneal specialist who may have attempted to use an intensive protocol for controlling IOP that involved the use of multiple antiglaucoma drugs. In contrast, better IOP control should be more easily achieved in patients with less severe diagnoses, and our results showed that IOP control was more successful in the group with more severe glaucoma. In other words, patients who were categorized as belonging to Group 1 were primarily treated during the latter half of the 10-year study period, which means that a change in surgical practice and decision making may have occurred. A change in surgical practice would suggest that the observed improvement in the IOPs of Group 1 patients was actually due to earlier intervention more than to the possibility of a patient selection bias.

In conclusion, AGV implantation appears to be a viable option for controlling IOP in patients with preexisting glaucoma after penetrating keratoplasty (PKP). In addition, we found that early AGV implantation results in a higher rate of AGV implant survival and a better VA outcome compared with delayed AGV implantation without increasing the risk of graft failure. There is also a low incidence of severe postoperative complications with the notable exception of graft decomposition. However, graft failure remains a challenge in such patients.
